# Exhausting T Cells During HIV Infection May Improve the Prognosis of Patients with COVID-19

**DOI:** 10.3389/fcimb.2021.564938

**Published:** 2021-09-27

**Authors:** Hua-Song Lin, Xiao-Hong Lin, Jian-Wen Wang, Dan-Ning Wen, Jie Xiang, Yan-Qing Fan, Hua-Dong Li, Jing Wu, Yi Lin, Ya-Lan Lin, Xu-Ri Sun, Yun-Feng Chen, Chuan-Juan Chen, Ning-Fang Lian, Han-Sheng Xie, Shou-Hong Lin, Qun-Fang Xie, Chao-Wei Li, Fang-Zhan Peng, Ning Wang, Jian-Qing Lin, Wan-Jin Chen, Chao-Lin Huang, Ying Fu

**Affiliations:** ^1^ Department of Neurology, The Second Affiliated Hospital, Fujian Medical University, Quanzhou, China; ^2^ Department of Neurology of First Affiliated Hospital, Fujian Medical University, Fuzhou, China; ^3^ Department of Tuberculosis and Respiratory Disease, Jin Yin-tan Hospital, Wuhan, China; ^4^ Department of Infectious Diseases, Jin Yin-tan Hospital, Wuhan, China; ^5^ Department of Clinical Laboratory, Jin Yin-tan Hospital, Wuhan, China; ^6^ Department of Radiology, Jin Yin-tan Hospital, Wuhan, China; ^7^ Department of Respiratory and Critical Care Medicine, The Second Affiliated Hospital, Fujian Medical University, Quanzhou, China; ^8^ Department of Intensive Care Medicine, The Second Affiliated Hospital, Fujian Medical University, Quanzhou, China; ^9^ Department of Respiratory Medicine of First Affiliated Hospital, Fujian Medical University, Fuzhou, China; ^10^ Department of General Medicine of First Affiliated Hospital, Fujian Medical University, Fuzhou, China; ^11^ Department of Gastroenterology, The Second Affiliated Hospital, Fujian Medical University, Quanzhou, China; ^12^ Department of Emergency Medicine, The Second Affiliated Hospital, Fujian Medical University, Quanzhou, China; ^13^ Department of Thyroid and Breast Surgery, The Second Affiliated Hospital, Fujian Medical University, Quanzhou, China; ^14^ Department of Thoracic Surgery, Jin Yin-tan Hospital, Wuhan, China; ^15^ Institute of Neuroscience, Fujian Medical University, Fuzhou, China

**Keywords:** COVID-19, HIV, T cells, T-cell exhaustion, lymphocyte redistribution

## Abstract

T-cell reduction is an important characteristic of coronavirus disease 2019 (COVID-19), and its immunopathology is a subject of debate. It may be due to the direct effect of the virus on T-cell exhaustion or indirectly due to T cells redistributing to the lungs. HIV/AIDS naturally served as a T-cell exhaustion disease model for recognizing how the immune system works in the course of COVID-19. In this study, we collected the clinical charts, T-lymphocyte analysis, and chest CT of HIV patients with laboratory-confirmed COVID-19 infection who were admitted to Jin Yin-tan Hospital (Wuhan, China). The median age of the 21 patients was 47 years [interquartile range (IQR) = 40–50 years] and the median CD4 T-cell count was 183 cells/μl (IQR = 96–289 cells/μl). Eleven HIV patients were in the non-AIDS stage and 10 were in the AIDS stage. Nine patients received antiretroviral treatment (ART) and 12 patients did not receive any treatment. Compared to the reported mortality rate (nearly 4%–10%) and severity rate (up to 20%–40%) among COVID-19 patients in hospital, a benign duration with 0% severity and mortality rates was shown by 21 HIV/AIDS patients. The severity rates of COVID-19 were comparable between non-AIDS (median CD4 = 287 cells/μl) and AIDS (median CD4 = 97 cells/μl) patients, despite some of the AIDS patients having baseline lung injury stimulated by HIV: 7 patients (33%) were mild (five in the non-AIDS group and two in the AIDS group) and 14 patients (67%) were moderate (six in the non-AIDS group and eight in the AIDS group). More importantly, we found that a reduction in T-cell number positively correlates with the serum levels of interleukin 6 (IL-6) and C-reactive protein (CRP), which is contrary to the reported findings on the immune response of COVID-19 patients (lower CD4 T-cell counts with higher levels of IL-6 and CRP). In HIV/AIDS, a compromised immune system with lower CD4 T-cell counts might waive the clinical symptoms and inflammatory responses, which suggests lymphocyte redistribution as an immunopathology leading to lymphopenia in COVID-19.

## Introduction

Lymphopenia is a common feature in patients with severe coronavirus disease 2019 (COVID-19), with drastically reduced numbers of T cells (Chen et al., 2020). A retrospective study of 522 patients with COVID-19 found that a clinical severity-dependent reduction in the number of T cells is inversely correlated with the serum level of interleukin 6 (IL-6) (Diao et al., 2020). This suggests that, as the disease severity progresses in patients with COVID-19, a concomitant rise in the inflammatory cytokine levels may drive the depletion of T-cell populations, which contributes to the host’s weaker defense and to worse recovery. Therefore, treatments boosting immunity are needed (Moon, 2020; Zheng et al., 2020).

However, the immune system is also affected by damaged lung cells through the release of cytokines, which can cause T cells to migrate to the lungs and lead to lymphopenia in the peripheral blood. Most patients with severe COVID-19 exhibit substantially elevated serum levels of pro-inflammatory cytokines such as IL-6 and IL-1β. Also, C-reactive protein (CRP) is found to be abnormally high (Jin et al., 2020). Pathological findings of COVID-19 patients showed a massive infiltration of lymphocytes in the lungs. Spleen atrophy was also observed (Cao, 2020). These findings indicate that aggressive lymphocyte redistribution in the lungs was induced and that treatments to regulate immunity are needed for COVID-19 infection (Huang et al., 2020; Mehta et al., 2020; Qin et al., 2020; Wan et al., 2020; Wu and Yang, 2020).

Defining the role of T-cell changes in COVID-19 patients provides potential targets for clinical management. HIV/AIDS naturally served as a T-cell exhaustion disease model for recognizing how the immune system works in the course of COVID-19 infection. Thereof, in this study, we conducted a comprehensive evaluation of the clinical and radiological features of COVID-19 patients with confirmed HIV infection to reveal the role of T lymphocytes in this disease.

## Materials and Methods

### Patients

On March 4, 2020, 38 suspected cases with fever and dry cough were transferred to Jin Yin-tan Hospital, considering their shared history of exposure to patients with confirmed COVID-19. Of them, 24 have HIV/AIDS. Subsequently, 21 patients were confirmed to be infected with COVID-19, the diagnosis of which was based on clinical characteristics, chest imaging, test of COVID-19 virus, and the ruling out of common bacterial and viral pathogens that cause pneumonia.

Initial investigations included complete blood count, T lymphocyte count, coagulation profile, and serum biochemical test. Respiratory specimens were tested for common viruses using real-time RT-PCR assays approved by the China Food and Drug Administration. Routine bacterial and fungal examinations were also performed.

### CT Visual Quantitative Evaluation

Two radiologists blinded to the clinical information reviewed all the images independently. The distribution and the density of lesions in each lobe, as well as the overall lung “total severity score” (TSS), were recorded. Each of the five lung lobes was assessed for percentage of lobar involvement and classified into none (0%), mild (<50%), or severe (>50%), with corresponding scores of 0, 1, and 2, respectively. The density in each lobe was classified as having ground-glass opacities or with mixed ground-glass opacities or consolidation, with corresponding scores of 1 and 2, respectively. The TSS was reached by summing the five lobe distribution scores multiplied by the density score (ranging from 0 to 20).

### Statistical Analysis

For variables that were non-normally distributed or are non-parametric, differences were assessed using the Mann–Whitney or Wilcoxon signed-rank test. Categorical variables were compared for the groups using the chi-square test (Fisher’s exact test when the expected value is <5). Statistical analysis was performed with SPSS 25 (IBM Corp., Armonk, NY, USA).

## Results

### Demographic and Clinical Characteristics

The median age of the 21 patients was 47 years [interquartile range (IQR) = 40–50 years]; there was a prevalence of females (2 males and 19 females). The HIV infection stages of these patients according to the WHO classification are as follows: 1 patient had no adverse event (CD4 > 500), 2 patients were mild (350 < CD4 < 499), 7 patients were advanced (200 < CD4 < 349), and 10 patients were severe (CD4 < 200) (**Table 1**). Considering the clinical features, 11 patients were non-AIDS and 10 were AIDS presenters according to the Centers for Disease Control and Prevention (CDC) classification. Nine patients received antiretroviral treatment (ART) and 12 patients did not receive any ART. During hospitalization for COVID-19, antiretroviral therapy with lamivudine and tenofovir was administered to 20 patients, except for one patient who refused, whose CD4 T-cell count was 85 cells/μl.

**Table 1:** Baseline, treatments, and outcomes of COVID-19 patients with HIV/AIDS.

	All (*N* = 21)	Non-AIDS (*N* = 11)	AIDS (*N* = 10)	*p*-value
Age (years)				0.338
<30	0 (0)	0 (0)	0 (0)	
30–40	6 (29)	4 (36)	2 (20)	
41–60	13 (62)	6 (55)	7 (70)	
>60	2 (10)	1 (9)	1 (10)	
CD4^+^ T-cell count				<0.001
>500	1 (5)	1 (9)	0 (0)	
351–500	2 (10)	2 (18)	0 (0)	
201–350	7 (33)	7 (64)	0 (0)	
100–200	5 (24)	0 (0)	5 (50)	
<100	5 (24)	0 (0)	5 (50)	
ART	9 (43)	6 (55)	3 (30)	0.466
Current smoking	6 (29)	1 (9)	5 (50)	0.063
Any comorbidity				
Hypertension	4 (19)	1 (9)	3 (30)	0.310
Diabetes	0 (0)	0 (0)	0 (0)	1.000
Cardiovascular disease	2 (10)	1 (9)	1 (10)	1.000
Chronic obstructive	0 (0)	0 (0)	0 (0)	1.000
Malignancy	0 (0)	0 (0)	0 (0)	1.000
Pulmonary disease	2 (10)	1 (9)	1 (10)	1.000
Chronic liver disease	2 (10)	2 (18)	0 (0)	1.000
COVID-19 type				0.362
Mild	7 (33)	5 (45)	2 (20)	
Moderate	14 (67)	6 (55)	8 (80)	
Severe—critical	0 (0)	0 (0)	0 (0)	
Signs and symptoms				
Highest temperature (°C)				1.000
>39	0 (0)	0 (0)	0 (0)	
38–39	0 (0)	0 (0)	0 (0)	
37.3–37.9	5 (24)	3 (27)	2 (20)	
Cough	8 (38)	4 (36)	4 (40)	1.000
Diarrhea	0 (0)	0 (0)	0 (0)	1.000
Dyspnea	3 (14)	1 (9)	2 (20)	0.586
White blood cell count, 10^9^/L				0.086
<4	10 (48)	3 (27)	7 (70)	
4–10	11 (52)	8 (73)	3 (30)	
>10	0 (0)	0 (0)	0 (0)	
Lymphocyte count, 10^9^/L				0.149
>1.0	16 (76)	10 (91)	6 (60)	
<1.0	5 (24)	1 (9)	4 (40)	
Hemoglobin (g/L)				
>110	14 (67)	8 (73)	6 (60)	0.659
<110	7 (33)	3 (27)	4 (40)	
Platelet count,10^9^/L				0.476
>100	20 (95)	11 (100)	9 (90)	
<100	1 (5)	0 (0)	1 (10)	
Aspartate aminotransferase (U/L)				1.000
>40	7 (33)	3 (27)	4 (40)	
<40	14 (67)	8 (73)	6 (60)	
Creatine kinase (U/L)				1.000
>185	0 (0)	0 (0)	0 (0)	
<185	21 (100)	11 (100)	10 (100)	
Procalcitonin (ng/ml)				
<0.1	20 (95)	10 (91)	10 (100)	1.000
0.1–0.5	1 (5)	1 (9)	0 (0)	
>0.5	0 (0)	0 (0)	0 (0)	
CRP (mg/L)				0.403
<2	16 (76)	9 (82)	7 (70)	
2–10	4 (19)	2 (18)	2 (20)	
>10	1 (5)	0 (0)	1 (10)	
IL-6 (pg/ml)				1.000
<10	19 (90)	9 (82)	10 (100)	
10–30	2 (10)	2 (18)	0 (0)	
>30	0 (0)	0 (0)	0 (0)	
ARDS	0 (0)	0 (0)	0 (0)	1.000
Oxygen support				1.000
No	20 (95)	10 (91)	10 (100)	
Nasal cannula	1 (5)	1 (9)	0 (0)	
Treatment				
Antiviral therapy	20 (95)	11 (100)	9 (90)	1.000
Antibiotic therapy	9 (43)	3 (33)	6 (67)	0.387
Use of corticosteroids	0 (5)	0 (100)	1 (10)	1.000
Prognosis				1.000
Hospitalization	0 (0)	0 (0)	0 (0)	
Discharge	21 (100)	11 (100)	10 (100)	
Death	0 (0)	0 (0)	0 (0)	

Data are n (%).

ART, antiretroviral therapy; CRP, C-reactive protein; ARDS, acute respiratory distress syndrome.

The clinical severity of COVID-19 in the 21 patients with HIV/AIDS was classified as follows: mild in 7 (33%) (five in the non-AIDS group and two in the AIDS group) and moderate in 14 (67%) (six in the non-AIDS group and eight in the AIDS group). This is according to the Guidance for Corona Virus Disease 2019 (6th edition) released by the National Health Commission of China (**Table 1** and **Supplementary Table S1**). Only one patient, who has congenital heart disease, needed nasal cannula oxygen support to correct hypoxemia (SPO_2_ = 93%). All patients were discharged after a mean hospital stay of 23 days.

### CT Evaluation

**Figure 1:**
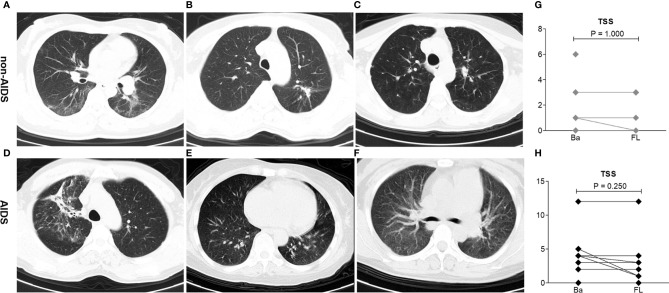
Imaging lung manifestations of coronavirus disease 2019 (COVID-19) patients with HIV/AIDS. **(A–C)** Representative chest CT of three patients with the most severe imaging in the non-AIDS group. **(A)** A 42-year-old woman had fever, cough, and sputum with a body temperature of 37.4°C. The CD4 T-cell count of this patient, who has received antiretroviral treatment (ART), was 263 cells/μl. Chest CT on the first day after admission demonstrated bilateral peripheral ground-glass opacities with linear opacities. The total severity score (TSS) was 5. **(B)** A 36-year-old woman had cough with a body temperature of 36.4°C. The CD4 T-cell count of this patient, who has not received ART, was 695 cells/μl. Chest CT on the first day after admission demonstrated peripheral ground-glass opacities with minimal consolidation in the left lung. The TSS was 3. **(C)** A 39-year-old woman had cough with a body temperature of 36.7°C. The CD4 T-cell count of this patient, who has not received ART, was 227 cells/μl. Chest CT on the first day after admission demonstrated peripheral minimal ground-glass opacities in the left lung. The TSS was 1. **(D–F)** Representative chest CT of three patients with the worst imaging in the AIDS group. **(D)** A 48-year-old man had cough and dyspnea with a body temperature of 36.4°C. The CD4 T-cell count of this patient, who refused any ART, was 85 cells/μl. Chest CT on the first day after admission demonstrated bilateral peripheral ground-glass opacities with minimal consolidation. The TSS was 12. **(E)** A 64-year-old woman had fever and cough with a body temperature of 37.6°C. The CD4 cell count of this patient, who received ART, was 95 cells/μl. Chest CT on the first day after admission demonstrated bilateral peripheral ground-glass opacities with minimal consolidation, mixed with HIV-infected lung imaging manifestations (nonspecific interstitial pneumonitis feature). The TSS was 10. **(F)** A 41-year-old woman had mild cough with a body temperature of 36.4°C. The CD4 T-cell count of this patient, who received ART, was 64 cells/μl. Chest CT on the first day after admission demonstrated bilateral peripheral ground-glass opacities, mixed with HIV-infected lung imaging manifestations (nonspecific interstitial pneumonitis feature). The TSS was 4. **(G, H)** Comparison of the TSS on admission (Ba) and on the day of follow-up (*FL*) after 20 days in hospital among non-AIDS and AIDS patients. Wilcoxon signed-rank tests were performed for each analyte.

On admission, all patients showed abnormalities in chest CT images. Of the 21 patients,14 (67%) had involvement and 8 (38%) had bilateral lesions. The most severe chest CT images in three patients each from the non-AIDS and AIDS groups are shown in **Figures 1A–F**. The imaging features were not completely consistent with previous literature reports on COVID-19 patients, which were mixed with HIV-infected lung imaging manifestations, especially in the AIDS group, such as a feature of chronic nonspecific interstitial pneumonitis (**Figures 1E, F**). Before discharge from the hospital, the second chest CT images of all patients did not show deterioration. The TSS of non-AIDS patients decreased from a median of 1.0 (range = 0–6) to 0 (range = 0–3), while the TSS of patients with AIDS decreased from a median of 3.5 (range = 0–12) to 2 (range = 0–12) (**Figures 1G, H**).

### T-Lymphocyte Analysis

**Figure 2:**
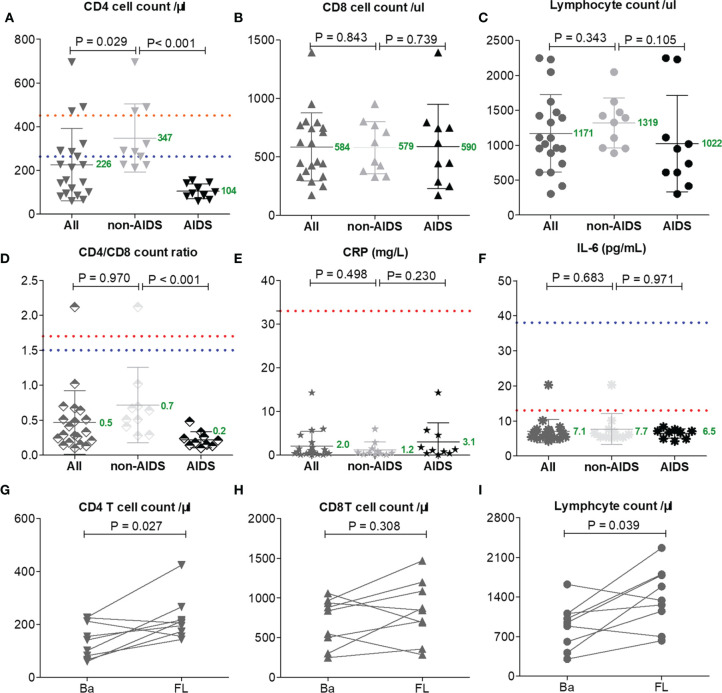
Lymphocyte subset counts and C-reactive protein (CRP) and interleukin 6 (IL-6) levels compared between AIDS and non-AIDS patients **(A–F)**. One patient had no lymphocyte subset data on admission and 12 patients lacked lymphocyte subset data at follow-up (FL) **(G–I)**. The Mann–Whitney test was performed for different group comparisons and Wilcoxon signed-rank test was performed for follow-up analysis. Mean values are shown in *green* for this study. *Orange* and *blue dotted lines* indicate the mean values reported by others for common patients or severe COVID-19 patients without HIV/AIDS (Qin et al., 2020).

T-lymphocyte subsets were analyzed in 20 patients on admission and in nine patients on the day before discharge from the hospital. The median CD4 T-cell count was183 cells/μl (IQR = 96-289 cells/μl) among 20 patients on admission; one patient lacked data on this. All HIV patients showed typical features of compromised immunity: the mean CD4 T-cell count (226 cells/μl) and the mean CD4/CD8 ratio (0.5) were reduced below the mean limits of the reported patients with either severe (CD4 = 263 cells/μl, CD4/CD8 = 1.5) or moderate (CD4 = 451 cells/μl, CD4/CD8 = 1.7) COVID-19 (Qin et al., 2020), and these were reduced more profoundly in AIDS than in non-HIV-infected COVID-19 cases (CD4 = 347 *vs*. 104 cells/μl, *p* < 0.001; CD4/CD8 = 0.7 *vs*. 0.2 cells/μl, *p* < 0.001, respectively) (**Figures 2A–D**).

The mean levels of CRP (2.0 mg/L) and IL-6 (7.0 pg/ml) in all HIV patients were markedly lower than the mean limits of the reported patients with either severe (CRP = 57.9 mg/L, IL-6 = 25.2 pg/ml) or moderate (CRP = 33.2 mg/L, IL-6 = 13.3 pg/ml) COVID-19 (Qin et al., 2020); the levels of CRP (1.2 *vs*. 3.1 mg/L, *p* = 0.230) and IL-6 (7.7 *vs*. 6.5 pg/ml, *p* = 0.971) among non-AIDS patients and those with AIDS were comparable (**Figures 2E, F**).

The lack of T cells in HIV patients had no impact on long-term virus control, and the discharge time of these patients was not prolonged. After 20 days of hospitalization, with clinical improvements, all patients had increased CD4 T cells and lymphocytes (*p* < 0.05; **Figures 2G–I**), which may have been caused by the antiretroviral therapy for HIV or immune activity in COVID-19 (Dessie et al., 2020).

## Discussion

T cells particularly play a significant antiviral role in combating pathogens. CD4 T cells have an important role in promoting the production of virus-specific antibodies by activating T-dependent B cells and enhancing CD8 T-cell toxicity in order to kill virus-infected cells. It has been reported that COVID-19 is more likely to infect older men with comorbidities (Huang et al., 2020), who have weaker immune functions. Several studies have shown that decreased CD4 T-cell and lymphocyte counts are associated with severe clinical types, which supports having weaker immune functions with T-cell exhaustion as contributing to the severity of COVID-19 (Moon, 2020; Zheng et al., 2020). However, in this study, we investigated 21 HIV/AIDS patients and found that all of them had a benign clinical form of COVID-19 and had better prognosis. Regardless of the CD4 cell counts and whether ARTs have been administered (**Table 1**), none of the patients progressed to the severe form or to death, despite some of the AIDS patients presenting with baseline lung injury stimulated by HIV (**Figure 1**).

The T-cell counts were significantly reduced in COVID-19 patients—the surviving T cells in severe COVID-19 patients with CD4 T-cell counts were lower than 400 cells/μl (Moon, 2020; Zheng et al., 2020)—but which were still higher than those of patients with HIV/AIDS in this study (226 cells/μl). COVID-19 caused a respiratory disease that led to a mortality rate of nearly 4%–10%: up to 20%–40% of patients required ventilatory support and even intensive care. However, the severity and mortality rates in this study were the lowest compared to previous reports, which indicates that a compromised immune system with a lower CD4 T-cell count might waive the clinical symptoms (Zhou F. et al., 2020).

HIV, as a T-cell exhaustion disease, leads to having a suboptimal immune function for defense against multiple viral infections. The lungs are one of the main target organs for virus-associated disease, and almost 70% of HIV patients suffer from at least one respiratory complication during the course of their illness (Rosen et al., 2001). In HIV patients, there is an enhanced prevalence of bronchial hyperresponsiveness and dysfunction of the small airways (Poirier et al., 2001). Surprisingly, when HIV patients are infected with COVID-19, they show mild symptoms. These data imply that the immune system actively participates in the severe pathogenesis of COVID-19. This immune activation may cause amplification of the inflammatory cascade and lead to a massive influx of lymphocytes to the lungs from the periphery (lymphocyte redistribution). Data reported on three aspects by others support this opinion: 1) accumulating evidence suggests that a subgroup of patients with severe COVID-19 might have higher serum levels of pro-inflammatory cytokines (TNF-α, IL-1, and IL-6) that activate T cells (Mehta et al., 2020; Qin et al., 2020; Wan et al., 2020; Wu and Yang, 2020); 2) pathological findings on the lungs of COVID-19 patients showed massive infiltration of lymphocytes in the lungs (Yang X et al., 2020); and 3) some chronic immune-inflammatory disease patients on immunosuppressants have no increased risk of COVID-19 (Brownlee et al., 2020; Monteleone and Ardizzone, 2020; Price et al., 2020).

Although immune-inflammatory treatment is not routinely recommended for COVID-19, according to the finding of this study and other findings about cytokine storm syndrome, a timely and appropriate use of immune modulators with antiretroviral therapy support should be considered for mild patients in order to prevent development of the severe form (Zhou Y. et al., 2020; Zhou et al., 2021). The sphingosine-1-phosphate receptor regulator fingolimod (FTY720) is an effective immunology modulator that targets CD4 T cells, which has been widely used in multiple sclerosis with a proven safety profile. Fingolimod acts as an immunology modulator that inhibits the egress of lymphocytes from the lymph nodes and limits their migration, especially CD4 T cells (Kappos L et al., 2010). With the hypothesis of reducing focal pulmonary inflammatory responses using fingolimod, this drug could be potentially considered for use in clinical trials.

## Conclusions

There are some caveats to the present study, such as the small sample size, its retrospective design, and the lack of detection of TCR zeta-chain expression (Baniyash, 2004). However, only a few clinical studies have, so far, tested the hypothesis that modulation of inflammation and immune response can ameliorate the severity of COVID-19. HIV/AIDS naturally served as a T-cell exhaustion disease model for us to recognize how the immune system works in the course of COVID-19. This study demonstrated that lower CD4 counts might waive the clinical symptoms and inflammatory responses in COVID-19, which would advance our understanding of the virus–immune system interaction in clinical settings. In addition, this study suggests that patients with chronic immune-inflammatory diseases (e.g., multiple sclerosis and rheumatic arthritis) should stay on immunosuppressant or immune modulator medications. On the other hand, it is noteworthy that suppression of the effector CD4 T lymphocyte could be beneficial for mitigating the progress of COVID-19.

## Data Availability Statement

The raw data supporting the conclusions of this article will be made available by the authors, without undue reservation.

## Ethics Statement

The studies involving human participants were reviewed and approved by Jin Yin-tan Hospital Ethics Committee (KY-2020-51.01). The patients provided written informed consent to participate in this study.

## Author Contributions

YF, and C-LH had full access to all of the data in the study and take responsibility for the integrity of the data and the accuracy of the data analysis. YF and C-LH contributed to the study concept and design. All authors acquired, analyzed, and interpreted the data. YF and X-HL drafted the manuscript. NW, W-JC and C-LH critically revised the manuscript for important intellectual content. YF did the statistical analysis, obtained funding, and provided administrative, technical, or material support. YF and C-LH supervised the study. All authors contributed to the article and approved the submitted version.

## Funding

This work has been supported by grant 81771279 (to YF) from the National Natural Science Foundation of China. This work also has been supported by grant No.2021MP03 (H-SL) from the Nursery Fund Project of the Second Affiliated Hospital of Fujian Medical University.

## Conflict of Interest

The authors declare that the research was conducted in the absence of any commercial or financial relationships that could be construed as a potential conflict of interest.

## Publisher’s Note

All claims expressed in this article are solely those of the authors and do not necessarily represent those of their affiliated organizations, or those of the publisher, the editors and the reviewers. Any product that may be evaluated in this article, or claim that may be made by its manufacturer, is not guaranteed or endorsed by the publisher.
