# Supplementary material for: Exhausting T Cells During HIV Infection May Improve the Prognosis of Patients with COVID-19
Source: Front Cell Infect Microbiol. 2021 Sep 27;11:564938. doi: 10.3389/fcimb.2021.564938 (PMC8502810; doi:10.3389/fcimb.2021.564938)
Supplement: Supplementary file 1 [file DataSheet_1.pdf]

Supplementary materials

e-Table 1. Criteria for clinical severity of confirmed coronavirus disease 2019 (COVID-19) *	
**Types**	**Definition**
**Mild**	Mild clinical symptoms [fever <38℃ (quelled without treatment), with or without cough, no dyspnea, no gasping, no chronic disease] No imaging findings of pneumonia
**Moderate**	Fever, respiratory symptoms, imaging findings of pneumonia
**Severe**	Meet any of the followings:<br>• Respiratory distress, RR ≥30 times/min<br>• SpO_2 <93% at rest<br>• $PaO_2/FiO_2 \leq 300$mmHg<br>• d. Patients showing a rapid progression (>50%) on CT imaging within 24-48 hours
**Critical**	Meet any of the followings:<br>• Respiratory failure, need mechanical assistance<br>• Multiple organ failure, intensive care unit (ICU) is needed<br>• Shock
RR = respiratory rate; SpO2: oxygen saturation; PaO2: partial pressure of oxygen; FiO2: fraction of inspired oxygen.<br>*according to the Guidance for Corona Virus Disease 2019 (6th edition) released by the National Health Commission of China	
